# Modality of wound closure after total knee replacement: are staples as safe as sutures? A retrospective study of 181 patients

**DOI:** 10.1186/1754-9493-5-26

**Published:** 2011-10-19

**Authors:** Justin T Newman, Steven J Morgan, Gustavo V Resende, Allison E Williams, E Mark Hammerberg, Michael R Dayton

**Affiliations:** 1 The Department of Orthopaedic Surgery, University of Colorado School of Medicine, Denver, Colorado USA; 2 Swedish Medical Center, Englewood, Colorado, USA; 3 The Department of Orthopaedic Surgery, Denver Health Medical Center, University of Colorado School of Medicine, Denver, Colorado, USA; 4 Bay Pines VA Health Care System, Bay Pines, Florida, USA

**Keywords:** wound closure, sutures, staples, total knee arthroplasty, complications

## Abstract

**Background:**

Surgical site wound closure plays a vital role in post-operative success. This effect is magnified in regard to commonly performed elective procedures such as total knee arthroplasty. The use of either sutures or staples for skin re-approximation remains a contested subject, which may have a significant impact on both patient safety and surgical outcome. The literature remains divided on this topic.

**Methods:**

Two cohorts of patients at a level one trauma and regional referral center were reviewed. Cohorts consisted of consecutive total knee arthroplasties performed by two surgeons who achieved surgical wound re-approximation by either staples or absorbable subcuticular sutures. Outcome variables included time of surgery, wound dehiscence, surgical site infection per Center for Disease Control criteria and repeat procedures for debridement and re-closure.

**Results:**

181 patients qualified for study inclusion. Staples were employed in 82 cases (45.3% of total) and sutures in 99 cases (54.7%). The staples group had no complications while the sutures group had 9 (9.1%). These consisted of: 4 infections (2 superficial, one deep, one organ/space); three patients required re-suturing for dehiscence; one allergic type reaction to suture material; and one gout flare resulting in dehiscence. The mean surgical time with sutures was 122.3 minutes (sd = 33.4) and with staples was 114 minutes (sd = 24.4).

**Conclusion:**

This study demonstrated significantly fewer complications with staple use than with suture use. While all complications found in this study cannot be directly attributed to skin re-approximation method, the need for further prospective, randomized trials is established.

## Background

Sutures and staples are applied interchangeably by surgeons in the skin closure of many types of wounds. It is widely accepted that both sutures and staples can achieve the basic goals of wound closure: a watertight, tension free non-inverted opposition of the skin edges that promotes rapid healing and a cosmetically acceptable scar[1]. Multiple studies have produced conflicting results regarding the efficacy, economics, rate of complications and cosmetic outcomes that are achieved when comparing these two closure methods for a variety of applications. A review of the literature reveals a paucity of information regarding closure of wounds after elective orthopaedic procedures and only two minor studies have specifically analyzed the impact about the knee, with no statistically significant differences found[2,3]. Discrepancies exist among current reports and no consensus exists to provide evidence based reasoning to guide orthopaedic surgeons to employ a specific type of skin closure technique. The purpose of this retrospective study was to compare staple skin closure to suture skin closure in patients undergoing primary total knee arthroplasty (TKA) with the intention of comparing surgical time and complications related to the closure technique. The hypothesis of the study is that no significant difference in clinical outcomes exists with regard to sutures or staples for skin closure after primary total knee arthroplasty.

## Methods

Following approval from the Institutional Review Board, we performed a retrospective review of the medical charts of two simultaneous and consecutively operated cohorts of patients who underwent primary total knee arthroplasty (TKA) from April 2003 to August 2006 at our facility. All patients admitted for primary TKA were eligible for inclusion in the review. Patients undergoing revision arthroplasty, those with an underlying malignancy, who had suffered previous trauma or who had a previous incision in the operative field were excluded.

All surgical procedures were performed by two of the authors of the paper (SM and MD). Surgical wound care and closure were performed without knowledge that the patients and their outcomes would be included in a study. All post-operative interventions undertaken were standard of care for our facility and were per the standardized protocol that was identical for each set of patients, including wound dressing and their removal, deep vein thrombosis prophylaxis and post-operative rehabilitation. Wound closure for all cases was performed with absorbable and non-absorbable suture at the level of the arthrotomy, and absorbable suture in the subcutaneous layer. The skin was re-approximated with either staples or a running subcuticular absorbable suture with steri-strips placed. Patient selection for either sutures or staples was based solely on treating surgeon's predilection for either sutures or staples. Cases varying from surgeon's routinely used closure method were due to ancillary staff preparing the non-standard method and that method being used in order to prevent waste. These patients were included in the final analysis as all primary knee arthroplasties were included in the analysis.

Information regarding skin closure was obtained from the operative record and verified with post-operative and clinical documentation. The duration of surgery was obtained from the operative record and was defined from time of initial incision until the skin closure was completed. A post hoc power analysis was then performed to determine the magnitude of the difference in surgical time between the two treatment groups. The patients' medical charts and clinic notes from follow-up visits were then reviewed for complications including: wound dehiscence, surgical site infection (SSI), repeat operations for debridement and re-closure, and documentation of tissue reaction to the closure material.

SSI was classified per the Center for Disease Control (CDC) criteria. Classifications of infections were: superficial/incisional, defined as involving only skin and subcutaneous tissue of the incision; deep incisional defined as involving peri-incisional deep soft tissues (e.g., fascial and muscle layers); and organ/space defined as involving any part of the body, excluding the skin incision, fascia, or muscle layers, that was opened or manipulated during the operative procedure.

Wound dehiscence was defined as primary if it occurred independently of an infection and secondary if it occurred as a sequela of an infection or other complicating process. Similarly, a repeat attempt at wound closure by re-suturing or re-stapling was defined as primary if it occurred independently of another procedure and secondary if it was after a planned procedure, such as a debridement.

Data were analyzed using SPSS 11.5 (SPSS, Chicago, IL). Univariate statistics including frequencies, percentages, and measures of central tendency were calculated for each measurement as well as for the demographic and injury characteristics. Mann Whitney testing was used for analysis when comparing demographic variables and outcomes between surgeons.

## Results

One hundred eighty-one primary TKAs were included in this analysis. One hundred and eighty four patients charts were reviewed. One patient was excluded due to a history of malignancy for which she had recently received chemotherapy treatment. Two patients were found to have had multiple operations secondary to trauma to the knee in question and were subsequently excluded. All patients had follow-up through closure of surgical wounds and resolution of surgical complications as demonstrated at follow-up clinic visits beyond the first pre-operative visit. (Table 1)

**Table 1:** Demographic Data and Complications in the Two Study Cohorts

	Number of cases	Mean age (standard deviation)	Complications	Superficial infections	Simple wound dehiscence	Deep soft tissue infection	Joint infection	Other	Mean total surgical time (Standard deviation)
Sutures Group	99	58.8 (8.8)	9	2	3	1	1	1 Gout flare resulting in dehiscence, 1 allergic reaction to suture material	122.3 minutes (33.4)

Staples group	82	59.9 (7.9)	0						114 minutes (24.2)

Eighty-two (45.3%) procedures were closed using staples and 99 (54.7%) using sutures. Surgeon SM performed seventy-nine (43.6%) of the cases and used staples in 76 cases (96.2% of his cases) and sutures in 3 cases. Surgeon MD performed 102 (56.4%) of the cases and used sutures in 96 cases (94.1% of his cases) and staples in 6 of the cases. All cases of sutures used Monocryl (poliglecaprone-25) as the closure method.

One hundred thirty (71.8%) patients were female and 51 (28.2%) were male. The average age of patients in the suture group was 58.8 years (sd = 8.8) and in the staples group 59.9 years (sd = 7.9). The average age of patients operated on by SM was 59.5 years (sd = 7.9). Surgeon MD's patients had a mean age of 59.1 years (sd = 8.8).

Surgical time for the suture group averaged 122.3 minutes (sd = 33.4) and 114 minutes (sd = 24.2) for the staples group. No significant differences were found between the suture and staple groups for surgical time or age (z = -1.198, p = 0.231 and z = -0.666, p = .505, respectively). Surgical time for SM averaged 117 minutes (sd = 24.0) and for MD 119.8 minutes (sd = 33.7). No significant differences were found between surgeons for surgical time or age (z = -0.107, p = 0.915 and z = -0.243, p = 0.808, respectively). The retrospective power analysis indicated a small effect size for surgical time by treatment group (current power 0.463 and d = 0.288). Given alpha = 0.05 and power = 0.80, approximately 390 subjects would be required to detect this effect (195 per group).

Nine persons (9.1%) in the suture group and no persons in the staples group developed a complication. The association between technique and incidence of a complication was statistically significant (χ^2 ^= 7.845, p = 0.004).

Complications consisted of 2 superficial infections, one deep infection, one organ/space infection that was a joint space infection and three cases of primary wound dehiscence without evidence of infection that required revision closure. There was one case of an allergic reaction to suture material in a patient who subsequently reported a history of hypersensitivity to suture material. This local tissue reaction resolved upon removal of suture material. One patient with a history of gout developed persistent drainage after his TKA procedure and was taken to the operating room for washout and exchange of polyethelyene insert, there was no clinical or culture evidence of infection and fluid analysis demonstrated crystals consistent with gout.

Of the reported infections, all occurred in the suture group, n = 4 (4%). In the group that was treated by surgeon SM, there was 1 infection. This was a superficial infection. In the group treated by surgeon MD, there were three infections in patients whose skin wounds were closed by sutures. These consisted of one superficial infection, one deep infection and one organ/space infection.

Primary wound dehiscence occurred in 3 (3.7%) patients in the suture group and no patients in the staple group. Secondary dehiscence occurred in 1 patient in the suture group and in no patients in the staple group. Four (4.9%) patients in the suture group required a repeat operation for debridement and re-closure, one was attributed to gout and three were secondary to infection.

## Discussion

This comparative study demonstrated a significant increase in surgical wound complications when skin closure was undertaken with suture methods in patients undergoing TKA. No complications were noted when skin closure was achieved with staple closure.

The strengths of the study include a relatively uniform randomization of patients to treating surgeon as consultations in our facility were made to the clinic and not to a specific surgeon. The patients are also presented in a consecutive series. The retrospective nature of this study lends credibility to the results in that it is an accurate representation of the care that was given. At the time procedures were performed the surgeons were unaware that the results would be analyzed and reported, effectively eliminating observer or participant bias that may occur with prospective trials that incorporate procedures.

The greatest weakness of this study is found in the predominant use of one method of wound closure by each of the surgeons. This introduces individual surgeon technique into the comparison of complications. Thus, we are unable to definitively discern whether differences in complications or surgical time were due to technique, surgeon or an interaction between the two variables.

The retrospective, comparative cohort study design also introduces a number of potential weaknesses. These include possible inconsistencies in documentation regarding wound complications and surgical times. The retrospective design does not allow for the accuracy and specificity of prospectively acquired data. In addition, a number of other potential confounding variables could not be accounted for or controlled including but not limited to the effect of body habitus, unrecognized or unrecorded medical conditions that may have compromised wound healing abilities, tourniquet time and drain use.

Focusing on the strengths of the study and understanding the inherent weaknesses of the evaluation, we feel it is reasonable to implicate the superficial infections and wound dehiscence in the suture group to the skin closure method.

The number of complications in this study appear to be higher than those found in a number of reports in the literature, however higher rates of complications have been recently reported when analyzing TKA surgical wound closure methods [2]. The higher rate found here may be due to a number of factors, including our analysis of all reported complications, irrespective of the likelihood of their being directly attributable to the closure method.

Automatic skin staplers were introduced for widespread use in 1972 with purported ease of usability and as a technique to reduce operative time[4,5]. Follow-up studies focusing on surgical time suggested that staples could save up to 80% of the time required for suturing with equal cosmetic results[6]. Two comparative studies from 1987 and 1992 reported faster wound closure time with staple use but at the cost of wound inflammation, discomfort, and diminished cosmetic results in laparotomy and general wound closure[7,8]. This was echoed in 1997 when it was reported that when closing pediatric scalp lacerations, staples were faster and more economical than sutures[9].

The literature in orthopedic surgery related to wound closure methods is mixed in its analysis. In a prospective randomized comparative study of sutures versus staples in skin closure of 66 hip surgery procedures, the only noted difference between techniques was better cosmesis with sutures[10]. Khan et al in 2006 compared subcuticular suture, skin staples and 2-octylcyanoacrylate for closure of wounds after hip and knee replacements and found similar results with both sutures and staples[2]. Singh et al found significantly less wound discharge and redness with the use of sutures for wound closure after surgery for fracture of the neck of the femur[11]. Further supporting the conclusion of these preceding studies in the field of cardiovascular surgery, it has been suggested that suturing was less expensive and associated with a decreased incidence of infection and inflammation when compared to stapling[12,13]. The present study did not look at the cosmetic effects of either technique but focused on the rate of wound complications associated with either technique and does not support the concept of suture superiority for closure of TKA. The difference between this study and that reported by Singh may be related to the location on the extremity. It is possible that in areas with a redundant blood supply and adequate soft tissues, like the hip, that closure method may be irrelevant. In the soft tissues surrounding the knee, particularly the area overlying the proximal tibia and patella, there may be a greater degree of vascular compromise based on wound closure technique.

Biologically friendly closure techniques may prevent peri-operative wound problems. Graham et al reported that staple use provided better blood perfusion to the wound site than sutures, which the authors correlated to improved conditions for wound healing[3]. This may explain the potential absence of wound issues in the staple group seen in this study.

## Conclusion

This study showed a significant increase in complications when sutures were used instead of staples for wound closure after primary total knee arthroplasty. Not all of the complications may be directly attributable to wound closure at the level of the skin. However, the overall significant difference found may be detrimental to patient safety. The need for future prospective, randomized and adequately powered trials is established.

## Competing interests

The authors declare that they have no competing interests.

## Authors' contributions

JN performed the chart review, data collection and contributed to the writing of the manuscript. SM contributed to the study design and to the writing of the manuscript. GR assisted in the chart review and data collection. AW contributed to the study design, performed statistical analysis and contributed to the writing of the manuscript. EH assisted in writing and formatting the article in style and content for publication. MD contributed to the study design and to the writing of the manuscript. All authors have read and agree with final manuscript.

## References

[B1] Shetty AA Kumar VS Morgan-Hough C Georgeu GA James KD Nicholl JE Comparing wound complication rates following closure of hip wounds with metallic skin staples or subcuticular vicryl suture: a prospective randomised trial J Orthop Surg (Hong Kong) 2004 12 2 191 193 10.1177/230949900401200210 15621905

[B2] Khan RJ Fick D Yao F Tang K Hurworth M Nivbrant B Wood D A comparison of three methods of wound closure following arthroplasty: a prospective, randomised, controlled trial J Bone Joint Surg Br 2006 88 2 238 242 10.1302/0301-620X.88B2.16923 16434531

[B3] Graham DA Jeffery JA Bain D Davies P Bentley G Staple vs. subcuticular vicryl skin closure in knee replacement surgery: a spectrophotographic assessment of wound characteristics Knee 2000 7 4 239 243 10.1016/S0968-0160(00)00055-7 11104916

[B4] Steichen FM Ravitch MM Mechanical sutures in surgery Br J Surg 1973 60 3 191 197 10.1002/bjs.1800600307 4571416

[B5] Lennihann RMM A comparison of staples and nylon closure in varicose vein surgery Vasc Surg 1975 9 4 200 203 1106030 10.1177/153857447500900403

[B6] Meiring L Cilliers K Barry R Nel CJ A comparison of a disposable skin stapler and nylon sutures for wound closure S Afr Med J 1982 62 11 371 372 7112304

[B7] Stockley I Elson RA Skin closure using staples and nylon sutures: a comparison of results Ann R Coll Surg Engl 1987 69 2 76 78 3566131 PMC2498348

[B8] Ranaboldo CJ Rowe-Jones DC Closure of laparotomy wounds: skin staples versus sutures Br J Surg 1992 79 11 1172 1173 10.1002/bjs.1800791122 1467895

[B9] Kanegaye JT Vance CW Chan L Schonfeld N Comparison of skin stapling devices and standard sutures for pediatric scalp lacerations: a randomized study of cost and time benefits J Pediatr 1997 130 5 808 813 10.1016/S0022-3476(97)80025-X 9152292

[B10] Clayer M Southwood RT Comparative study of skin closure in hip surgery Aust N Z J Surg 1991 61 5 363 365 10.1111/j.1445-2197.1991.tb00235.x 2025190

[B11] Singh BMMGNSM Closure of hip wound, clips or subcuticular sutures: does it make a difference? EJOST 2006 16 2 124 129 10.1007/s00590-005-0043-2 28755123

[B12] Chughtai T Chen LQ Salasidis G Nguyen D Tchervenkov C Morin JF Clips versus suture technique: is there a difference? Can J Cardiol 2000 16 11 1403 1407 11109037

[B13] Trick WE Scheckler WE Tokars JI Jones KC Reppen ML Smith EM Jarvis WR Modifiable risk factors associated with deep sternal site infection after coronary artery bypass grafting J Thorac Cardiovasc Surg 2000 119 1 108 114 10.1016/S0022-5223(00)70224-8 10612768

